# Morningside Asylum, Edinburgh

**Published:** 1892-10-22

**Authors:** 


### The Institutional Workshop.

### WITHIN THE ASYLUMS.

### MORNINGSIDE ASYLUM, EDINBURGH.

By Our Special Commissioner.

The State Legislature of New York has, we understand, enacted that henceforth all institutions for the treatment of lunatics under its jurisdiction, shall be denominated hospitals for the insane. We could wish that it were possible in this country to abolish the word "asylum" altogether in favour of "hospitals for the insane." The history of insanity shows how the world has progressed since the days when lunatics were treated as dangerous nuisances, and oppressed, tormented, and neglected in dens unfit for human occupation. The madman's later period of imprisonment becomes one of open doors and bright surroundings, often with an abundance of healthy exercise and work, features which now combine to form the best type of a modern institution devoted to the care of the insane. By all means let us get into our own minds and endeavour to impress on the public that lunacy is a disease which is often capable of treatment resulting in ultimate cure. It would not be a bad idea for Parliament to insert a clause in the next Lunacy Act, providing that all establishments for the insane should be denominated hospitals.

These views, which have been forced upon us by a prolonged study of the many questions having reference to lunatics and their treatment, were emphasised by a visit we recently paid to Morningside, under Dr. Clouston's most intelligent and instructive guidance. Twenty years ago Morningside contained many of the worst features formerly met with in these establishments. Structurally many parts of it were abominable. All this has been changed, and at the present time, though no doubt it is a sad sight to see many of the cases under treatment, still the predominant feeling in the visitor's mind is one of thankfulness, for the whole atmosphere and appearance of the place is cheery, bright, and hopeful. Indeed, he meets in all directions with practical evidence that the work here done is of the best kind, and produces the most fruitful and beneficial results.

Of course, and not unnaturally, where the majority of the buildings at a public institution are old and unattractive, it is usual to advocate their demolition, and the erection of a new structure replete with every modern improvement. Dr. Clouston has, however, added to his many useful services that of a painstaking and successful reconstruction of the old buildings at Morningside at a relatively small cost. His plan has been to take the old buildings, to make a plan of them, to study this plan closely, and then, with the aid of an intelligent architect, to see how, and in what manner, it was possible by taking down corridor walls and throwing the spaces into day-rooms, by building out towers and bow windows, and by raising and adding ornamental roofs, to convert a dismal prison into a bright, attractive residence, which even the staff, despite their difficult duties, feel loth to leave or remove from. No one can go through Morningside as it is at present without being conscious of the value of continuous effort on the part of a medical superintendent to vary every part of a hospital for the insane, both as to its structure and also as to the style of its decorations and furniture. Apart altogether from the patients and their treatment, it is most helpful to an intelligent man to go about his duties day by day in any building every part of which presents varied and special features of an attractive character. The unconscious relief which such surroundings offer to mind and body cannot be too strongly emphasised.

It has been said that asylums are at present often too elaborately decorated and furnished. This idea has been started by so-called economists, whose relatively small acquaintance with practical details leads them to conclude, that coldness and repulsion are cheap as they are certainly nasty, while artistic attractiveness is luxuriant and costly. As a matter of fact, speaking generally, it may be declared with truth and force, that a medical superintendent who will take the trouble, as so many of our superintendents now do, to keep his eyes open, to study decoration, and to cultivate a taste for it, will be able with the aid only of the working staff which have necessarily to be maintained to effect the ordinary repairs and cleansing, to make his asylum decoratively so attractive as to raise a howl of criticism from the said economists, although the actual sum expended may be no greater (and in some cases we have known it to be even less) than the amount formerly spent upon whitewash and paint. In this connection we should like to say, that although we have noticed in the course of our inspections that here and there a superintendent may have succeeded in making his dormitories very artistic and attractive by the use of material in imitation of tapestry, still we think, on hygienic grounds alone, that it is undesirable to treat the walls of any apartment where a patient is to be placed after this manner. Every superintendent should strive to secure the best results without running any hygenic risks, such as those referred to. Again, there are yet superintendents who hold to the feeling that a day-room should be a secure apartment, the door of which can be closed and locked. They object altogether to corridors being utilised to increase the day-room space, and have a pious horror of breaking up patients into groups, and so adding materially to the comfort at any rate of some of their number. It is difficult to argue with those who hold such limited views. But the great experience of eminent specialists like Dr. Clouston and others convinces us that the well-being and comfort of the patients, as well as those of the asylum staff, are increased by the abolition of the plain, unattractive, secure, square day-room, where all the patients were equally uncomfortable. In Russia, though many of the institutions are uninteresting, they study internal decoration much more than we do, and to an extent which, to our western ideas is wonderful, not only on account of the beauty of the interiors of their palaces and houses, but from the infinite variety displayed. So we hold that those asylum superintendents, who, like Dr. Clouston, will take a pride and spare no trouble or personal effort to think out and design new features for every portion of the establishment under their control, do real public service, the influence of which will extend to posterity, and the results of which must lead to incalulable good.

### The New Craig House and Villas.

We went to Morningside after spending a day at the Royal Infirmary. The new nurses' home there, which will be by far the most elegant and complete building of its kind in this country, was designed by Mr. Mitchell, the same architect who the governors and managers of the Royal Edinburgh Hospital have employed for the new buildings. The elevation of New Craig House is remarkable from the fact that it is free from repetitions, that no commonplace reproduction is to be seen, and that the whole edifice presents a fine and attractive appearance whatever point it may be viewed from. In addition to the New Craig House, the Old Craig House has been modernised and made a most comfortable residence for a few patients, under the charge of a matron. The ingenious way in which the apartments of this old house—built so long ago as 1565—have been panelled, and otherwise adapted so as to make everyone of them attractive and pleasing is beyond all praise. The same may be said of the modern villas and detached hospitals which the managers have sanctioned as a part of the New Craig House scheme. One of the villas is already completed, and anyone, no matter what his position in life may be, will be able to learn from seeing it how much additional pleasure a house can be made to afford those who will take the trouble to study decorative art, and to carry it out in detail by making each room a special feature complete in itself. In the dining-room, for instance, the whole of the furniture is practically a reproduction of the best Chippendale design. The chairs are most handsome and comfortable, and yet Dr. Clouston has been able to provide them at a price which he would have had to give for a common dining-room chair of the most ordinary pattern. Even in the bed-rooms each has its own features, and from kitchen to rooftree everything is excellently conceived and admirably carried out. Downstairs and away from the part of the house occupied by the patients is a single-bedded room, where a troublesome or dirty patient can be placed so as to prevent him from being a nuisance or difficulty.

The New Craig House is not likely to be complete for another eighteen months, although it has been in course of erection since 1890. We shall hope to give a full account of this remarkable building when it is ready for occupation. Meanwhile, we may frankly say we are convinced that it will fulfil a useful purpose by the beneficial effects it is likely to have upon everyone who visits it. The most pronounced architectural feature will be the great hall, which must be entered in order to get to any portion of the buildings. Every room has been made a special study. Not to cumber this article with too many details, we will simply add, by way of illustration, that no two rooms contain a ceiling of the same design, and that every design has been specially worked out, and is entirely new. One of the principles laid down before the plans were drawn were adaptation of the various parts of this establishment to the varied needs and mental states of of its inhabitants. This means much variety carefully thought out. At least five kinds of accomodation are to be provided, viz., acute wards, convalescent wards, attached villas, hospitals, and detached villas. About half the patients will live outside the central building. Much pleasure must result to every person who visits the New Craig House, and Dr. Clouston and the architect are entitled to warm thanks and hearty congratulations. But we are of opinion that substantial recognition is also due to the governors and managers of the Royal Edinburgh Asylum for the wisdom they have displayed in sanctioning the erection of these buildings, which will mark a new departure likely to be memorable in the annals of medical institutions. Remembering the constitution of this Royal Hospital at Morningside, it is matter for the grateful recognition of the whole nation that so much wisdom should have been displayed by those who have had to administer the funds of this great charitable trust.

